# Spatiotemporal characteristics and primary influencing factors of typical dengue fever epidemics in China

**DOI:** 10.1186/s40249-019-0533-9

**Published:** 2019-03-28

**Authors:** Lan Zheng, Hong-Yan Ren, Run-He Shi, Liang Lu

**Affiliations:** 1 Key Laboratory of Geographic Information Science, Ministry of Education, East China Normal University, Shanghai, China; 2 State Key Laboratory of Resources and Environmental Information System, Institute of Geographic Sciences and Natural Resources Research, Chinese Academy of Sciences, Beijing, China; 3 School of Geographic Sciences, East China Normal University, Shanghai, China; 4 Joint Laboratory for Environmental Remote Sensing and Data Assimilation, East China Normal University and Institute of Remote Sensing and Digital Earth, Chinese Academy of Sciences, Shanghai, China; 5 Department of Vector Biology and Control, Chinese Center for Disease Control and Prevention, Natural Institute for Communicable Disease Control and Prevention, Beijing, China

**Keywords:** Spatiotemporal pattern, China, Dengue fever, Generalized additive model, Socioeconomic factor, Environmental factor

## Abstract

**Background:**

Dengue fever (DF) is a common mosquito-borne viral infectious disease in the world, and increasingly severe DF epidemics in China have seriously affected people’s health in recent years. Thus, investigating spatiotemporal patterns and potential influencing factors of DF epidemics in typical regions is critical to consolidate effective prevention and control measures for these regional epidemics.

**Methods:**

A generalized additive model (GAM) was used to identify potential contributing factors that influence spatiotemporal epidemic patterns in typical DF epidemic regions of China (e.g., the Pearl River Delta [PRD] and the Border of Yunnan and Myanmar [BYM]). In terms of influencing factors, environmental factors including the normalized difference vegetation index (NDVI), temperature, precipitation, and humidity, in conjunction with socioeconomic factors, such as population density (Pop), road density, land-use, and gross domestic product, were employed.

**Results:**

DF epidemics in the PRD and BYM exhibit prominent spatial variations at 4 km and 3 km grid scales, characterized by significant spatial clustering over the Guangzhou-Foshan, Dehong, and Xishuangbanna areas. The GAM that integrated the Pop-urban land ratio (ULR)-NDVI-humidity-temperature factors for the PRD and the ULR-Road density-NDVI-temperature-water land ratio-precipitation factors for the BYM performed well in terms of overall accuracy, with Akaike Information Criterion values of 61 859.89 and 826.65, explaining a total variance of 83.4 and 97.3%, respectively. As indicated, socioeconomic factors have a stronger influence on DF epidemics than environmental factors in the study area. Among these factors, Pop (PRD) and ULR (BYM) were the socioeconomic factors explaining the largest variance in regional epidemics, whereas NDVI was the environmental factor explaining the largest variance in both regions. In addition, the common factors (ULR, NDVI, and temperature) in these two regions exhibited different effects on regional epidemics.

**Conclusions:**

The spatiotemporal patterns of DF in the PRD and BYM are influenced by environmental and socioeconomic factors, the socioeconomic factors may play a significant role in DF epidemics in cases where environmental factors are suitable and differ only slightly throughout an area. Thus, prevention and control resources should be fully allocated by referring to the spatial patterns of primary influencing factors to better consolidate the prevention and control measures for DF epidemics.

**Electronic supplementary material:**

The online version of this article (10.1186/s40249-019-0533-9) contains supplementary material, which is available to authorized users.

## Multilingual abstracts

Please see Additional file 1 for translations of the abstract into the five official working languages of the United Nations.

## Background

Dengue fever (DF) is an acute infectious disease caused by the dengue virus, which is transmitted by *Aedes albopictus* and *Aedes aegypti* [1]. Approximately one-third of the global population is exposed to DF, which is widely endemic in tropical and subtropical areas, especially in Southeast Asia, the Western Pacific, and southern Africa [2]. In recent years, the increasing incidence and range of DF epidemics have had a serious impact on people’s health and lives, and DF has become a public health problem that should not be underestimated.

In the mainland of China, DF is currently a localized epidemic caused by imported cases. No case was reported in China from 1949 to 1977 until an outbreak occurred in Guangdong Province in 1978, since then, China’s DF epidemic has been intermittent [1]. With the acceleration of globalization and China’s increasingly frequent international exchanges, the prevalence of DF induced by imported cases has increased substantially [3–6]. In recent years, DF epidemics have frequently occurred not only in southern China [7–9] but also in some inland areas, such as Henan (Xuchang) and Shandong (Jining) [10]. Overall, China’s DF epidemic has shown increasingly shorter time intervals and a wider spread. In southern China, some typical regions with frequent DF epidemics have developed [11–14], especially in the Pearl River Delta (PRD) and the Border of Yunnan and Myanmar (BYM), and the local DF cases in the PRD and BYM accounted for 97.06% of cases nationwide from 2010 to 2014.

In the absence of effective vaccines, domestic and foreign scholars have conducted a large number of studies on factors, that affect the spread and prevalence of DF epidemics, such as the dengue virus, mosquito vectors, susceptible population, and environmental and socioeconomic factors [15–18]. Among these factors, environmental conditions, such as climate, hydrology, and vegetation, mainly affect the activity of the dengue virus, the breeding environment, and mosquito vector activity [19–21]. Socioeconomic factors, such as population density, land use, transportation convenience, residents’ income level, and living habits, play an important role in DF epidemics by changing both the probability of bites from the mosquito vectors and their activities [22–25]. Previous studies on the epidemic scale, spatiotemporal characteristics, and influencing factors of DF have deepened our understanding of DF characteristics in China [1, 19]. However, additional knowledge is needed regarding the spatial-temporal characteristics of the epidemic in China’s typical DF epidemic areas and the differences in influencing factors. Our study was performed to 1) analyse the spatial-temporal pattern of DF epidemics in two regions and 2) use a generalized additive model (GAM) to analyse and compare the main influencing factors affecting the spatial disparities of DF in the two regions. The results of this study will provide important support for strengthening the prevention and control of DF outbreaks in the PRD and BYM and raising the level of prevention of DF risk.

## Methods

### Study area

**Fig. 1:**
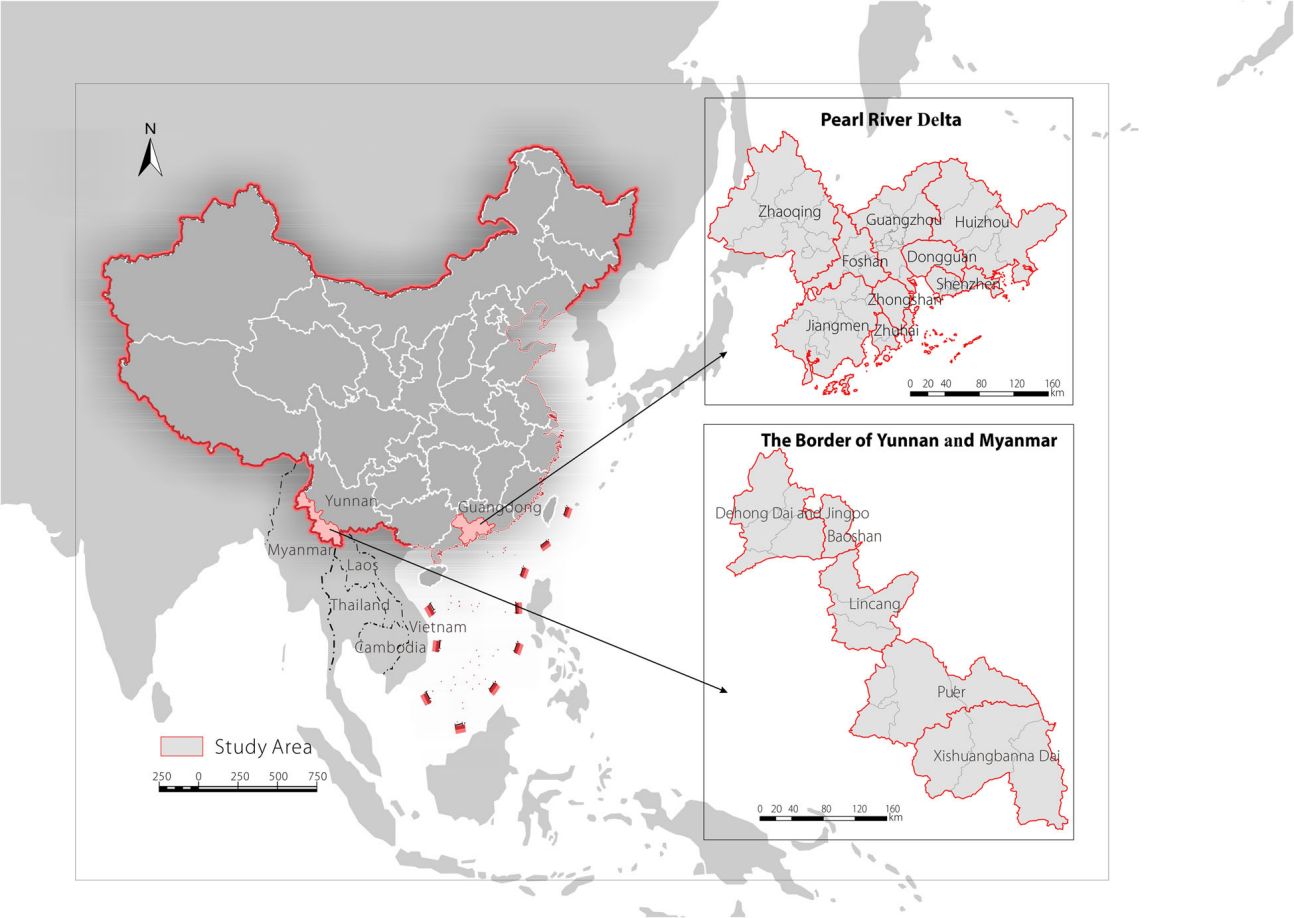
Illustration of the study areas

The PRD (111°28′–114°42′E, 22°16′–23°57′N) in the Guangdong Province is located at the Pearl River estuary and includes nine cities, such as Guangzhou and Foshan (Fig. 1). This area represents one of the major hubs for China’s economic growth and is one of the most urbanized regions in the world. In addition, the PRD has a high population of 58.74 million, and the gross domestic product (GDP) per capita was approximately RMB 107000 yuan in 2015. The PRD also has a subtropical monsoon ocean climate that is humid and warm and has abundant sunshine hours throughout the year [26].

The BYM (97°56′–101°34′E, 21°28′–24°43′N) referred to in our study mainly consists of Xishuangbanna, Dehong, and parts of Lincang and Pu’er in Yunnan Province. It is located on the boundary of Myanmar and Laos and presents several important international trade ports. In addition, this region has a population of approximately 4.4 million and a 2015 GDP per capita of approximately RMB 25000. Limited differences in temperature are observed throughout the year, although a large temperature difference occurs between day and night. The wet and dry seasons are distinct, and the water system in the area is developed.

### Data collection

#### DF incidence data

Records of observed DF cases from 2010 to 2014 were obtained from the China Notifiable Disease Surveillance System, and the data included age, gender, occupation, date of onset, and type of diagnosis. In this study, only local cases were used to analyse the spatial-temporal characteristics of the local epidemic and the relationship between the pattern of the epidemic and local variables (environmental and socioeconomic). The DF cases were spatially located with geocoding (http://www.gpsspg.com/xGeocoding/) to enable calculation of the number of DF cases on different spatial grid scales.

#### Environmental and socioeconomic data

**Fig. 2:**
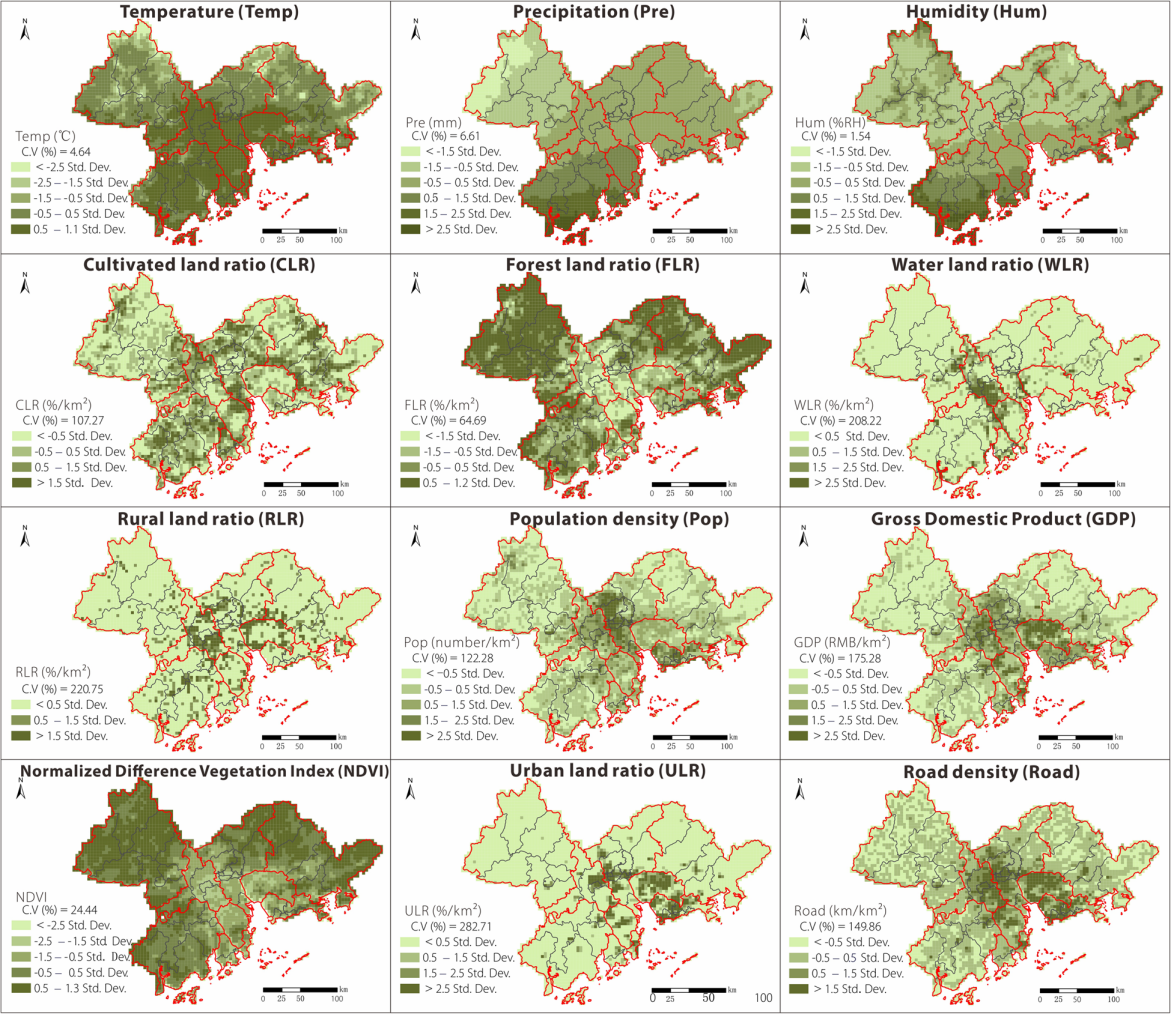
Spatial distributions of environmental and socioeconomic factors in the Pearl River Delta; grid scale of 4 km × 4 km

**Fig. 3:**
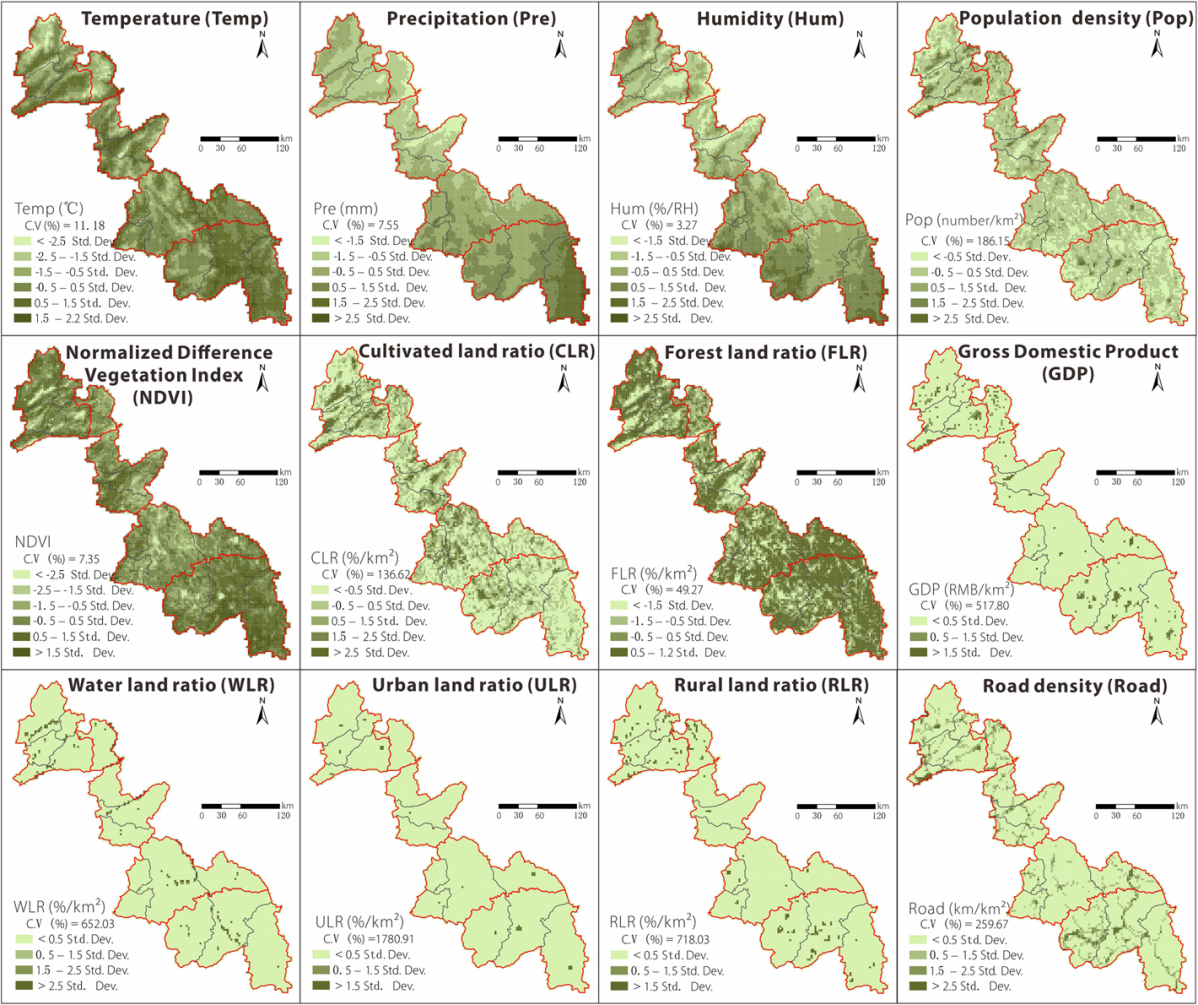
Spatial distributions of the environmental and socioeconomic factors in the Border of Yunnan and Myanmar; grid scale of 3 km × 3 km

In accordance with previous studies [21, 27–29], this study selected four environmental factors (mean temperature [Temp], mean relative humidity [Hum], mean precipitation [Pre], and normalized difference vegetation index [NDVI]) that temporally correspond to the epidemic data (from April to November). In addition, four socioeconomic variables in 2010 (land use data, population size, road density, and GDP) were obtained to reflect the regional social conditions. All variables (see Table 1 for details on data processing) were calculated from original data of 1 km^2^ resolution, and the spatial distribution of these factors is illustrated in Figs. 2 and 3.

**Table 1:** Data sources and processing of environmental and socioeconomic factors

Variables/description	Data processing	Data source
Mean temperature	Mean temperature from 2010 to 2014 (April–November) in each grid	China Meteorological Data Service Center (CMDC, http://data.cma.cn/)
Mean precipitation	Mean precipitation from 2010 to 2014 (April–November) in each grid	
Mean relative humidity	Mean relative humidity from 2010 to 2014 (April–November) in each grid	
Vegetation index	Mean normalized difference vegetation index (NDVI) from 2010 to 2014 (April–November) in each grid	https://ladsweb.modaps.eosdis.nasa.gov/
Land use	According to the land use coverage classification system of the Data Center for RESDC, land use data in 2010 were divided into cultivated land, forest land, grass land, water areas, urban land, rural residential areas, other construction land, and unused land. Statistics on the proportion of various land use areas in the grid	Resource and Environmental Science Data Center of the Chinese Academy of Sciences (RESDC, www.resdc.cn)
Population size	Summing the population (persons) for each grid based on the 2010 population density data	
Economic conditions	Summing the gross domestic product (GDP) values (RMB) for each grid based on the 2010 GDP data	
Road density	Summing the road density (km/km^2^) for each grid based on the road network data in 2010	OpenStreetMap (http://download.geofabrik.de/)

### Research unit

Basic geographic units, such as districts, counties, towns, and streets, are frequently altered by the constant changes in administrative divisions in epidemiological studies. However, this phenomenon can be effectively avoided by creating regular spatial grids [30]. A spatial autocorrelation analysis is often used to reflect the spatial aggregation of a feature in the region. In this study, a series of spatial grids (1 × 1 km–14 × 14 km) was created, and the optimal grid of the DF spatial pattern in each region was selected based on Moran’s *I* [31]. Moran’s *I* is expressed by Eq. (1) as follows:

1 $$ {Moran}^{\hbox{'}}s\;I=\frac{n\sum \limits_{i=1}^n\sum \limits_{j=1}^n{w}_{ij}\left({x}_i-\overline{x}\right)\left({x}_j-\overline{x}\right)}{\sum \limits_{i=1}^n\sum \limits_{j=1}^n{w}_{ij}\sum \limits_{i=1}^n{\left({x}_i-\overline{x}\right)}^2} $$

where *n* is the number of grids in the study area, *x*_*i*_ and *x*_*j*_ represent the number of DF cases in grids *i* and *j*, respectively, and *w*_*ij*_ is the matrix of spatial weight. Moran’s *I* is generally tested by the *Z*-score/*P*-value, and the value varies from − 1 to 1. A higher Moran’s *I* (larger *Z*-score and proper *P*-value) indicates greater similarity among attributes between adjacent spatial grids [32], which reveals that the DF epidemic is clustered in the region, whereas a low negative value indicates dissimilarity between adjacent grids and shows that the DF epidemic is discretely distributed in the region [33]. In this study, Moran’s *I* and *Z*-scores of the DF cases with different grid sizes were used to assess the optimal grid scales of the regional DF epidemic. Spatial autocorrelation analysis above was performed using ArcGIS 10.2 (ESRI, Redlands, CA, USA).

### Statistical analysis

The GAM is a semiparametric model extended from the generalized linear model [34, 35]. It can provide both linear and nonlinear fitting to variables, and it has been widely used in infectious epidemiology, such as for DF, in recent years [23, 35, 36]. The model automatically selects the appropriate polynomial by establishing the smoothing function of the independent variable and identifies and estimates the nonlinear optimality of the model from data.

2 $$ g\left(\mu \right)={\beta}_0+\sum {\beta}_i\left({X}_i\right)+\sum {S}_i\left({X}_i\right) $$

In Eq. (2), *g*(*μ*) denotes a link function that can select the corresponding link function according to the different statistical distributions of dependent variables. Consistent with previous studies, the distribution of DF cases in this study fits a Poisson distribution [23]. Thus, the corresponding link function for the GAM model is log(y). The variable y refers to the number of local DF cases in the grid from 2010 to 2014(log(*DF case*)), *β*_0_ is a constant term, *β*_*i*_(*X*_*i*_) represents the linear fitting function, and *Si*(*Xi*) represents the nonlinear fitting function. The independent variable *X*_*i*_ represents the 12 variables (ratio of land use area [cultivated land, forest land, water area, rural residential land, and urban land], population density [Pop], road density [Road], NDVI, GDP, Hum, Temp and Pre) under the optimal grid.

The first step is to build the single factor model by using the spline smoothing function of the GAM, and then the goodness of fit of single factors is statistically analyzed. Next, the variables that did not pass the significance test in the single factor analysis are removed. Then, variables with strong collinearity are sorted into groups, and one variable in each group and other variables without strong collinearity are selected to build the GAM until all permutations and combinations are considered. Finally, the optimal GAM is selected according to the Akaike information criterion (AIC), with a better model corresponding to smaller AIC values [21]. The spatial data processing was completed in ArcGIS 10.2 software, and all the statistical analyses were performed using the statistical software R 3.0.3 (Lucent Technologies, Jasmine Mountain, USA) with the mgcv library.

## Results

### Temporal and spatial distribution of DF

**Fig. 4:**
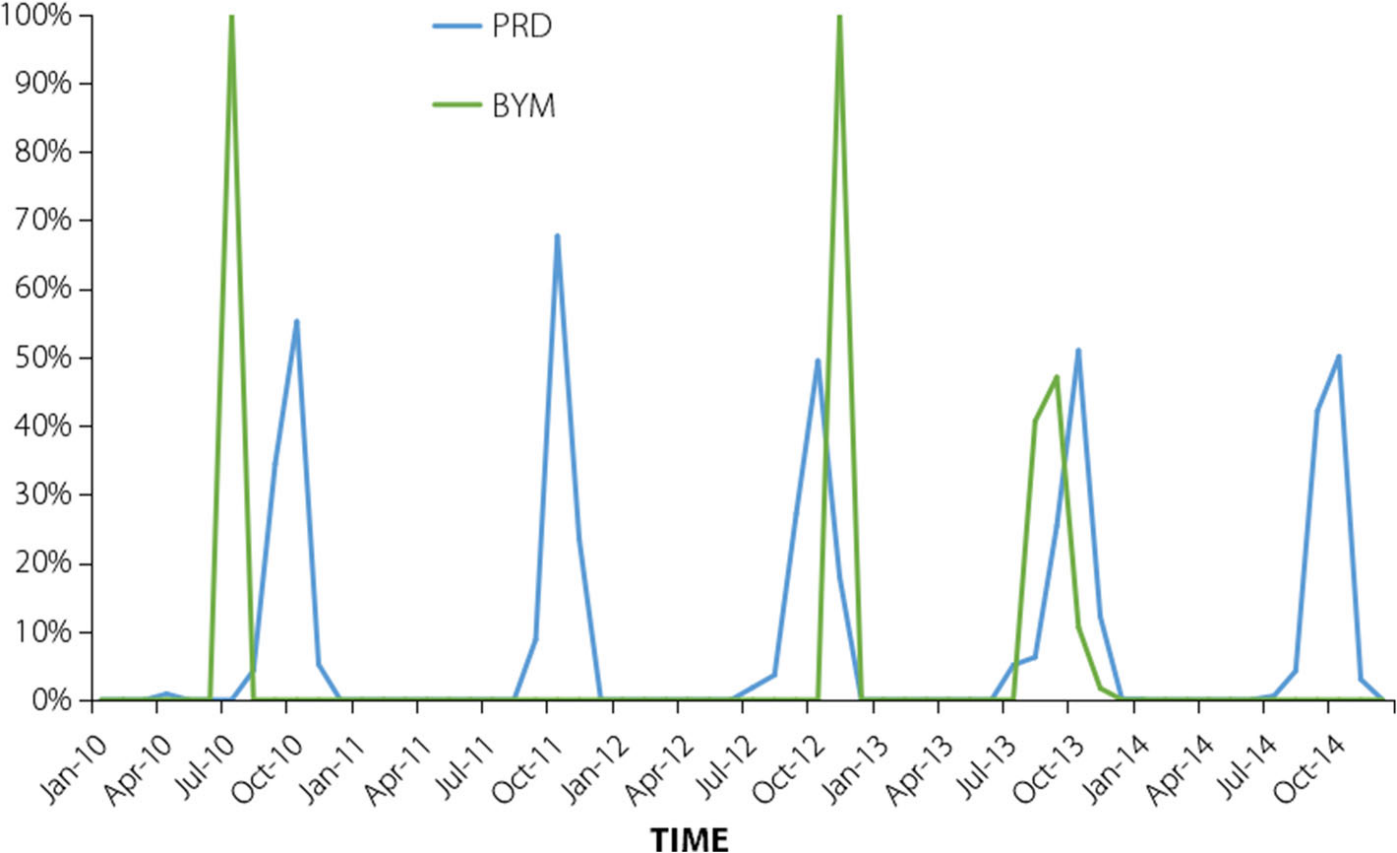
Ratio of monthly local cases to the total annual local cases in 2010–2014; PRD: Pearl River Delta; BYM: Border of Yunnan and Myanmar

According to the China Notifiable Disease Surveillance System, 49 290 local DF cases occurred in China between 2010 and 2014, with those in the PRD and BYM accounting for 97.06%. Figure 4 shows that the DF epidemic had obvious seasonal characteristics. The epidemic was mainly concentrated in July to November, accounting for 99.95% of the annual cases, and reached the peak incidence from August to October. In these 5 years, there were 46 422 and 1419 local DF cases in the PRD and BYM, respectively, showing an increasing trend in successive years. From 2010 to 2012, there were fewer than 500 local cases, and the number of cases in 2013 and 2014 surged to 4000 and 40 000, respectively, with the BYM exhibiting a large-scale epidemic in 2013.

**Fig. 5:**
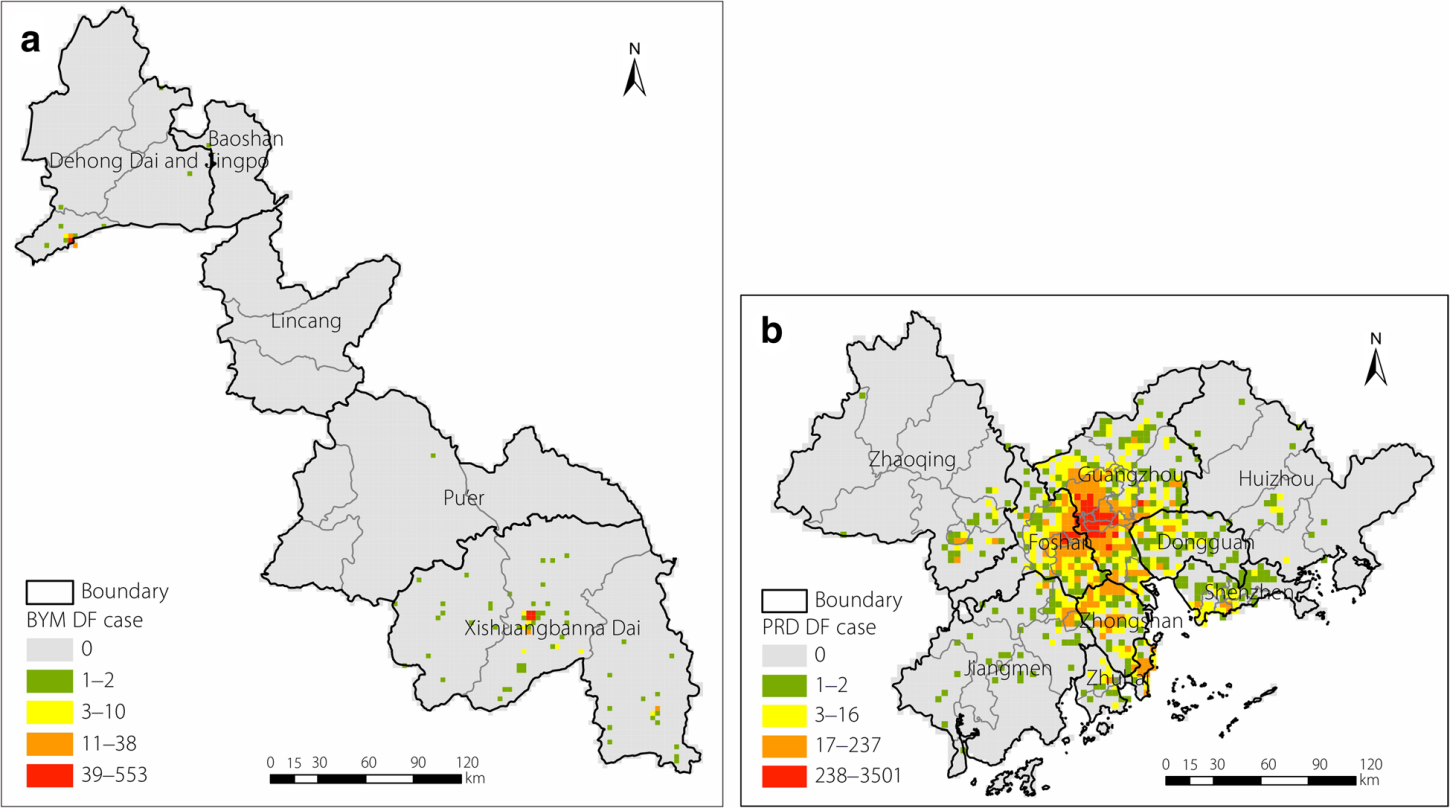
Spatial distribution of DF in the PRD and BYM; **a** BYM grids of 3 km × 3 km, and **b** PRD grids of 4 km × 4 km; DF: dengue fever; PRD: Pearl River Delta; BYM: Border of Yunnan and Myanmar

Table 2 lists the Moran’s *I* values of the DF cases at different grid scales in the PRD and BYM, which were calculated by Eq. (1). The PRD showed better clustering of DF epidemic cases at the 4 km × 4 km grid scale, whereas the BYM showed better clustering at the 3 km × 3 km scale. Furthermore, all Moran’s *I* values in the PRD were greater than those in the BYM, which indicated that the DF epidemic of the PRD was highly aggregated, while that of the BYM was relatively decentralized. In terms of the spatial distribution mapped in Fig. 5, the DF cases in the PRD presented an aggregative distribution cantered in the Guangzhou-Foshan region, whereas cases in the BYM were mainly concentrated in Dehong and Xishuangbanna Prefectures. These results demonstrate that DF cases in the BYM and the PRD showed significant characteristics of spatial aggregation.

**Table 2:** Spatial autocorrelation analysis of dengue fever cases in the Pearl River Delta and the Border of Yunnan and Myanmar from 2010 to 2014

	Gridded Scales	1 km	2 km	3 km	4 km	5 km	6 km	7 km	8 km	9 km	10 km	11 km	12 km	13 km	14 km
Pearl River Delta	Moran’s *I*	0.59	0.73	0.73	**0.77**	0.73	0.67	0.65	0.47	0.57	0.54	0.60	0.31	0.40	0.46
	*Z*-score	417.16	206.80	119.04	**69.81**	57.31	45.01	40.31	35.73	29.69	26.46	23.15	20.76	21.28	18.08
	*P*-value	< 0.01	< 0.01	< 0.01	**< 0.01**	< 0.01	< 0.01	< 0.01	< 0.01	< 0.01	< 0.01	< 0.01	< 0.01	< 0.01	< 0.01
Border of Yunnan and Myanmar	Moran’s *I*	0.15	0.23	**0.31**	0.18	0.27	0.27	0.11	0.01	0.31	0.27	0.22	0.27	0.01	0.12
	*Z*-score	90.17	54.62	**47.75**	20.57	26.88	22.26	20.79	3.74	16.59	13.63	11.98	12.11	2.10	10.44
	*P*-value	< 0.01	< 0.01	**< 0.01**	< 0.01	< 0.01	< 0.01	< 0.01	< 0.01	< 0.01	< 0.01	< 0.01	< 0.01	< 0.05	< 0.01

boldface: dengue fever cases showed significant characteristics on this grid scale

### GAM fitting

Pearson correlation coefficient analysis (see Additional files 2 and 3) and variable collinearity analysis (see Additional file 4) show that strong collinearity occurred in both the PRD (among the urban land ratio [ULR], road, and GDP; among Pop, Road, and GDP; and between the forest land ratio and NDVI) and the BYM (among Pop, ULR, and GDP; and between Hum and Pre). In each region, the model that passed the collinear diagnosis with the lowest AIC value was used as the optimal model for the DF epidemic to avoid over fitting of the model (see Additional file 4). Thus, the optimal GAM of the DF epidemic in the PRD consisted of five variables (Pop, ULR, NDVI, Hum, and Temp), and that in the BYM consisted of six variables (ULR, Road, NDVI, Temp, water land ratio [WLR], and Pre). The total variance of the GAM for the DF epidemic was 83.4% (R^2^ = 0.834, PRD) and 97.3% (R^2^ = 0.973, BYM), which shows that the GAM fit the regional differences of the epidemic well.

Regarding the variance explained by the single factor in the optimal GAM (see Additional file 4), socioeconomic factors explained more of the variance (> 54%) than environmental factors (< 54%). Among the socioeconomic factors, Pop and ULR explained the greatest variance in the DF epidemic in the PRD, whereas the ULR, followed by road density, explained the greatest variance in the DF epidemic in the BYM. For environmental conditions, NDVI ranked first in the two regions, followed by Hum-Temp (PRD) and Temp-WLR-Pre (BYM). These results show that the factors affecting the DF epidemic were generally similar but presented slight differences between the PRD and BYM.

### Comparison of the main DF factors in the two regions

**Fig. 6:**
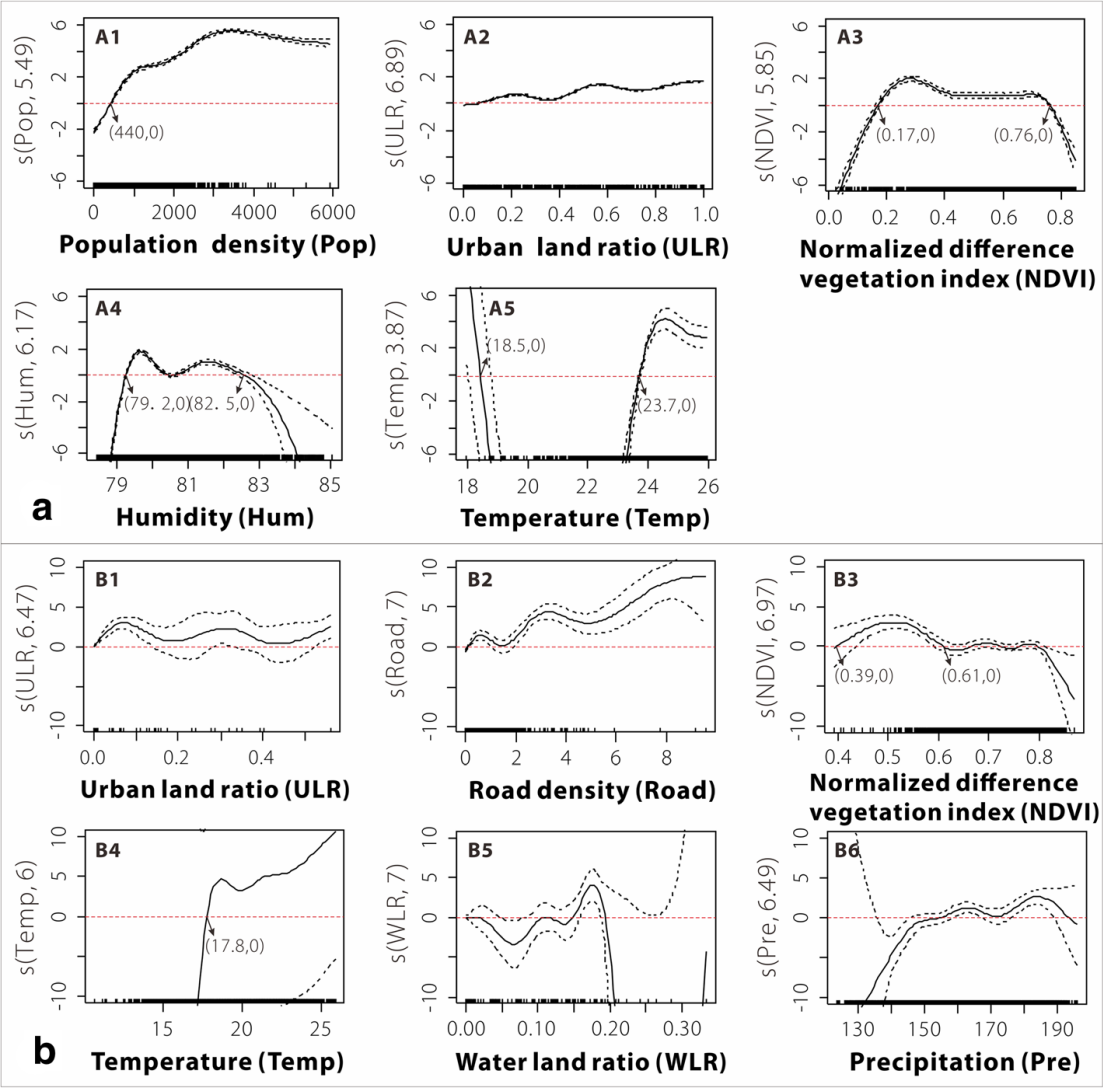
Generalized additive models-estimated relationships between dengue fever cases and influencing factors; **a** Pearl River Delta (PRD), A1: Population density (Pop), A2: Urban land ratio (ULR), A3: Normalized difference vegetation index (NDVI), A4: Humidity (Hum), A5: Temperature (Temp), **b** Border of Yunnan and Myanmar (BYM), B1, Urban land ratio (ULR), B2: Road density (Road), B3: Normalized difference vegetation index (NDVI), B4: Temperature (Temp), B5: Water land ratio (WLR), B6: Precipitation (Pre). The solid line shows the smooth fitting curve for the logarithm of dengue fever cases. The dashed line represents the 95% confidence intervals. The x-axis represents the actual values of the independent variables. The y-axis indicates the logarithm of dengue fever cases fitting values. Edf represents the estimated degrees of freedom. The y-axis is labelled s (a, edf), where a indicates the name of the variables and edf represents the estimated degrees of freedom of the smooth function, which is used to represent its relationship with dengue fever cases

As shown in Fig. 6, the nonlinear characteristics between the DF epidemic and the independent variables were obvious. In terms of the PRD, the DF epidemic was more serious in areas (grids) with a higher socioeconomic status, especially in the areas with 440 < Pop < 3500 (Fig. 6A1) and ULR > 0.4 (Fig. 6A2), while the DF epidemic tended to be stable in areas with a Pop > 3500 (Fig. 6A1). Compared with Pop and ULR, the nonlinear characteristics between environmental factors and the DF epidemic were more obvious. Among the factors, an ‘M’ relationship was observed between NDVI and the DF epidemic (Fig. 6A3), and the DF epidemic was serious when the NDVI was between 0.17 and 0.76, especially from 0.17 to 0.4. Similar to the NDVI, Hum also had an ‘M’ relationship with DF (Fig. 6A4), and the DF epidemic was relatively serious when Hum in the region was 79.2–82.5%. In contrast to the pattern observed for the above factors, the DF epidemic showed a clear “break” when the Temp of the region was 19–23 °C (Fig. 6A5), while the DF epidemic was more severe when Temp was below 18.5 °C (limited distribution) or above 23.7 °C (wider distribution).

Compared with the PRD, the DF epidemic in the BYM was relatively clustered in urban areas and showed gentle fluctuations with increases in ULR (Fig. 6B1). The ULR of the areas with relatively serious DF epidemics was approximately 0.07 and 0.3, and DF epidemics showed a wave rise as the road density increased (Fig. 6B2). Although the DF epidemic and the NDVI (ranking first among environmental factors) also showed an ‘M’ relationship in the BYM (Fig. 6B3), the NDVI value in relatively severe epidemic areas was 0.39–0.61. In addition, the DF epidemic was relatively serious in the BYM where the average Temp was higher than 17.8 °C (Fig. 6B4), the WLR was between 0.15 and 0.20 (Fig. 6B5), and the Pre was approximately 180 mm (Fig. 6B6). In general, the main factors (socioeconomic and environmental factors) and their nonlinear relationships with DF epidemics in the PRD and BYM were significantly different.

**Fig. 7:**
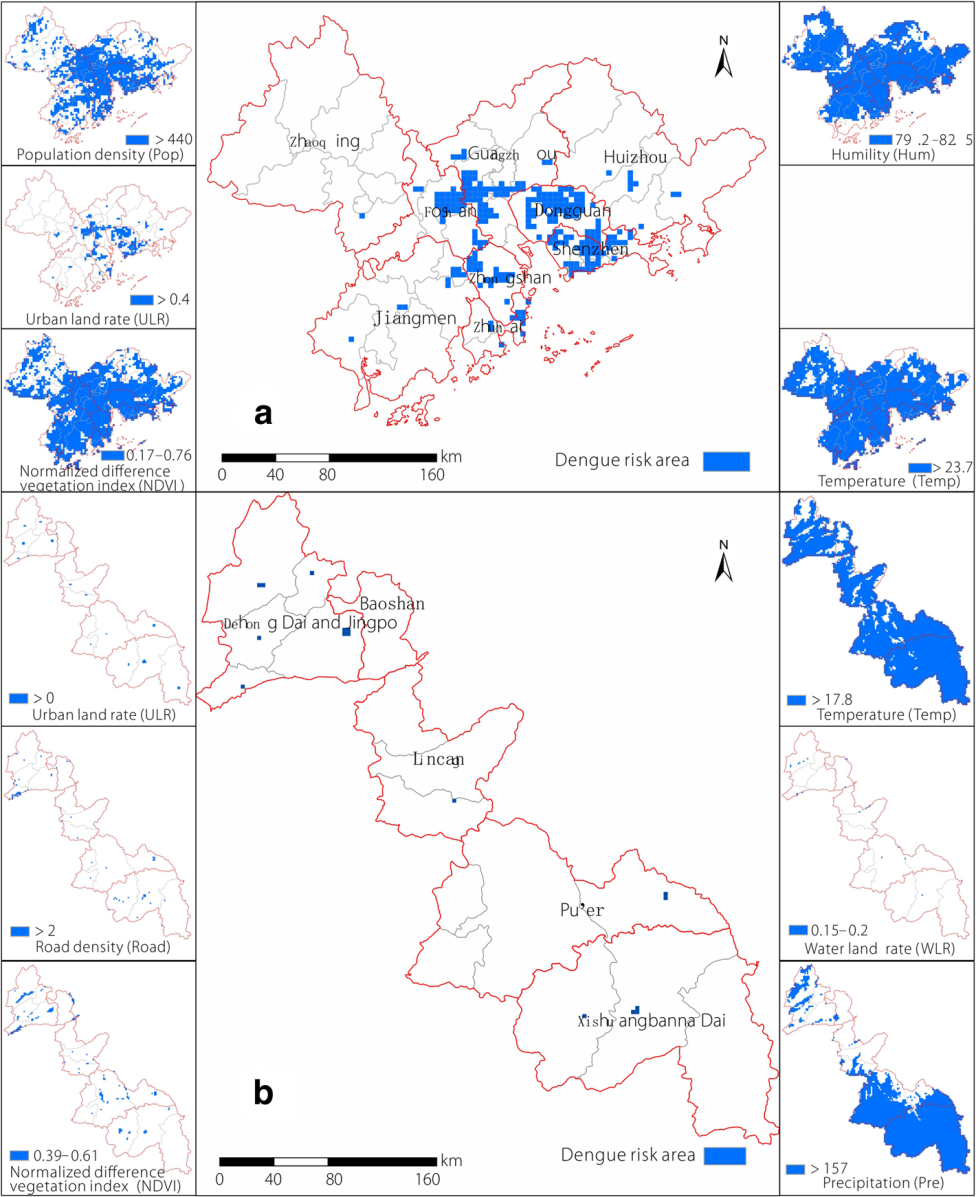
Dengue fever risk areas derived from the generalized additive models fitting results; **a** Pearl River Delta (PRD), and **b** Border of Yunnan and Myanmar (BYM)

In terms of spatial distribution, Guangzhou-Foshan, as well as Dongguan and Shenzhen, were areas with serious DF epidemics in the PRD (Fig. 7a). These areas appeared to have high ULR (> 0.4), high Pop (> 430), and moderate NDVI (0.17 < NDVI < 0.76). In addition, DF epidemics were more serious if the Hum was moderate (approximately 79.5% or 81.5%) and the Temp was higher than 23.7 °C. In comparison, DF epidemics in the BYM were relatively scattered throughout the Dehong and Xishuangbanna Prefectures (Fig. 7b). These areas have ULRs ranging from 0 to 0.5, developed road networks (road density > 2 km/km^2^), and moderate NDVIs (0.39 < NDVI < 0.61). DF epidemics were more acute in areas with Temp > 17.8 °C, Pre at moderate levels (170–190 mm), or water bodies (ratio of approximately 0.15–0.2).

## Discussion

In this study, we used the GAM to analyse and compare the main factors that affect the differences between DF epidemics in the PRD and BYM on the grid scale. Strengthening our understanding of the spatial-temporal patterns and differences between the influencing factors of DF epidemics in China’s typical DF epidemic areas is of considerable significance because such data can improve our ability to prevent and control the DF risk in high-incidence areas.

The epidemic characteristics of the PRD and BYM have quite a few similarities in environmental and socioeconomic factors. Regarding environmental conditions, previous studies have shown that suitable meteorological conditions (Temp at 20–30 °C and Hum of 75%) were conducive to mosquito breeding and reproduction [37, 38], thereby promoting dengue virus activity and increasing the risk of DF epidemics and transmission [39, 40]. According to the basic data of this study, the monthly mean Temp in the PRD and BYM is between 10 °C and 27 °C from April to November, the monthly mean Pre is 0–360 mm, and the relative Hum is 63–84%. These warm and humid weather conditions are conducive to DF transmission. In addition, the vegetation conditions in these two regions are good (the average NDVI is 0.57 to 0.81). Higher or lower NDVI values will reduce the risk of DF [41, 42]. High NDVI values generally indicate sparsely populated areas that are unable to meet the conditions of human and mosquito contact for DF. The appropriate vegetation conditions provide not only excellent conditions for the breeding and activity of mosquitoes (*Aedes albopictus* is primarily observed in the PRD, and *Aedes aegypti* is primarily observed in the BYM) [43] but also cool locations in summer for humans, thereby increasing the probability of contact between humans and mosquitoes and promoting DF epidemic risk [44]. The warm and humid environmental conditions in the BYM and PRD are suitable for mosquito breeding and activity, which is an important reason for the rapid and widespread prevalence of DF and presents the first similarity of DF epidemics in these two regions.

As for the second similarity of the characteristics of DF epidemic in these two regions, Ren et al. considered that socioeconomic factors might play a significant role in DF epidemics in cases where environmental factors were suitable and differed slightly in regional [13]. This finding is consistent with our results. Although the socioeconomic status (Pop, ULR, and Road) of the PRD is significantly greater than that of the BYM, these two regions present similar differences in development, and this finding can also be confirmed that the coefficient of variation of socioeconomic factors is significantly higher than that of environmental factors. In addition, Zhu et al. noted that a certain degree of population aggregation was an important condition for DF transmission and prevalence [45]. The population is relatively clustered in towns of the BYM (such as Dehong and Xishuangbanna) and the highly urbanized regions of the PRD (such as Guangzhou-Foshan). Therefore, this condition gives a reasonable explanation for the relatively serious epidemic in these areas. In recent years, the PRD has implemented active prevention and control measures, such as water retention and mosquito prevention, and DF epidemics have been effectively controlled. Therefore, based on the similarities between the two regions regarding the spatial patterns and main influencing factors underlying DF epidemics, we suggest that the BYM can draw on the experience of the PRD when formulating DF epidemic prevention and control strategies at the regional level.

Compared with the similarities listed above, the differences in the DF epidemic characteristics between the PRD and the BYM should be further investigated. Regarding the socioeconomic factors, previous studies have shown that a higher ULR corresponded to a larger population, while developed transport networks increased the mobility of people in the region [23, 24, 46, 47]. The extent and concentration of DF epidemics in the PRD are higher than those in the BYM, which is closely related to the higher Pop, higher ULR, uniform road network distribution, and stronger population mobility. The ULR of the BYM is high only in the central town areas of Dehong and Xishuangbanna (but still lower than that of the PRD), as are Pop and Road. The accessibility of roads throughout the region is not as good as that in the PRD, which can also explain why DF epidemics in the PRD are significantly greater than those in the BYM.

As for the regional environmental factors, the overall vegetation coverage in the BYM is relatively high. Therefore, the NDVI fitting curve of the more serious epidemic areas is shifted to the left compared with that in the PRD. Furthermore, the terrain within the BYM is complex, including large numbers of mountains and valleys. Therefore, regional differences and vertical changes in climate are obvious [48], which is different from the decreasing trend of temperature and precipitation in the PRD from south to north and from coast to inland [49]. The coefficient of variation of the environmental factors in the BYM is also slightly higher than that in the PRD. Meanwhile, the risk of DF tends to be higher in Dehong and Xishuangbanna Prefectures due to their higher annual average Temp and Pre, thus providing a favorable living environment for the *Aedes* mosquito, which is widely distributed throughout Ruili City (Dehong Prefecture) and Xishuangbanna Prefecture [50].

As for imported cases of DF, the BYM has several ports through which DF is mainly imported by “ground”, including Jinghong Port, Simao Waterway Port, and the busiest port of Rili to Myanmar. These wide ranges of trade ports promote local economic development but also increase the risk of imported DF cases because DF is highly prevalent in areas adjacent to Laos and Myanmar. In addition, Wang et al. confirmed that the local DF epidemic in the BYM in 2013 was caused by imported cases from the neighbouring countries of Southeast Asia [51]. The developed economy of the PRD results in the majority of imported DF cases being brought by relatively long-distance commercial travel. Meanwhile, as one of the most densely populated areas in China, more than one million migrant workers travel to the PRD each year. These people live in crowded and poor sanitary conditions and less rectified living areas, which are conditions conducive to human-mosquito contact [23, 46]. The BYM and PRD are both important ports of land and sea-air entry and exit in China, and they are popular locations for tourists and migrant workers in China. If DF epidemics develop explosive outbreaks in both regions without being effectively controlled, they will spread to adjacent inland areas, which will cause serious impacts to the life and health of people in inland areas. Therefore, the health departments must consider the spatial differentiation characteristics of the main factors affecting DF epidemics in the region and utilize these data to formulate more specific prevention and control strategies.

Several limitations are worth noting. (1) The difference in the severity of the two regional epidemics leads to a significant difference in the confidence interval of the fitting curve, although it does not affect the research paradigm. (2) The effects of mosquito vector and control measures have not been sufficiently considered, and these data should be included in our future work. (3) The spatial correlation between DF epidemics and various influencing factors has not been properly considered in the model, and models such as the GWR, which can consider such spatial correlations, should be included in future studies. (4) This study used the number of DF cases from 2010 to 2014 as the dependent variable, without carefully analysing the lagging effect and temporal effect between DF and its influencing factors.

## Conclusions

The environmental and socioeconomic factors in the PRD and BYM may affect the spatial-temporal differentiation of DF epidemics, and the influencing mechanisms have their own regional characteristics. The differences in socioeconomic factors are more obvious in cases where environmental factors are suitable and differ slightly throughout areas. This study has improved our understanding of the spatial distribution of DF epidemics and their influencing factors in typical regions of China. We suggest that the epidemic prevention and control strategies for the BYM should be developed in reference to those for the PRD, combined with the characteristics of the main factors influencing the regional epidemic to effectively strengthen the prevention and control measures for DF epidemics.

## Additional files

Additional file 1: Multilingual abstracts in the five official working languages of the United Nations. (PDF 225 kb)

Additional file 2: Pearson correlation analysis of variables in the PRD. (DOCX 19 kb)

Additional file 3: Pearson correlation analysis of variables in the BYM. (DOCX 18 kb)

Additional file 4: Statistical characteristics of variables and GAM fitting results. (XLSX 14 kb)

## Abbreviations

AIC: Akaike Information Criterion
BYM: The Border of Yunnan and Myanmar
CLR: Cultivated land ratio
DF: Dengue fever
ESRI: Environmental Systems Research Institute
FLR: Forest land ratio
GAM: Generalized additive model
GDP: Gross domestic product
Hum: Humidity
NDVI: Normalized difference vegetation index
Pop: Population density
PRD: Pearl River Delta of China
Pre: Precipitation
RLR: Rural land ratio
Road: Road density
Temp: Temperature
ULR: Urban land ratio
USA: United States of America
WLR: Water land ratio
