# Supplementary material for: Spatiotemporal characteristics and primary influencing factors of typical dengue fever epidemics in China
Source: Infect Dis Poverty. 2019 Mar 28;8:24. doi: 10.1186/s40249-019-0533-9 (PMC6440137; doi:10.1186/s40249-019-0533-9)
Supplement: Supplementary file 1 — Multilingual abstracts in the five official working languages of the United Nations. (PDF 225 kb) [file 40249_2019_533_MOESM1_ESM.pdf]

Translation of the abstract into the five official working languages of the United Nations

الخصائص الزمانية والمكانية وعوامل التأثير الرئيسة لأوبئة حمى الضنك في الصين

لان زينج، وهونج-يان رين، وران-هي شي

تمهيد

خلفية: حمى الضنك هي أكثر الأمراض الفيروسية المعدية التي ينقلها البعوض شيوعاً في العالم، وقد أثرت أوبئة حمى الضنك الشديدة أثراً كبيراً في صحة الناس في الصين خلال السنوات الأخيرة. ومن ثم، يُعَد التحقق من الأنماط الزمانية والمكانية لأوبئة حمى القش وعوامل التأثير المحتملة لها في مناطق معينة جوهرياً لترسيخ تدابير وقاية وضبط فعّالة ضد هذه الأوبئة المستوطنة.

أساليب أ كان النموذج الجمعي العام يُستخدَم للتعرف على العوامل المساهمة المحتملة التي تؤثر في أنماط الأوبئة الزمانية المكانية في مناطق في الصين التي تستوطن بها وباء حمى الضنك (على سبيل المثال: دلتا نهر اللؤلوة والحدود بين مقاطعة يونان وميانمار). فيما يتعلق بعوامل التأثير، وُظِفَت العوامل البيئية، بما فيها: مؤشر التغير الطبيعي للنباتات الخضراء، ودرجة الحرارة، ونسبة مياه الأمطار، والرطوبة، بالتزامن مع العوامل الاجتماعية الاقتصادية مثل: الكثافة السكانية، وكثافة الطرق، واستخدام الأرض والناتج المحلي الإجمالي.

النتائج: تُظهِر أوبئة حمى القش في دلتا نهر اللؤلؤة والحدود بين مقاطعة يونان وميانمار اختلافات مكانية بارزة على مستويات شبكية تصل إلى 3 و4 كيلومترات، وتتميز بتكتل مكاني بارز في مناطق كوانزو فوشان وديهونج وزيشوانجبانا. النموذج الجمعي العام الذي أدمج عوامل معدل الكثافة السكانية على الأرض العمرانية ومؤشر التغير الطبيعي للنباتات الخضراء والرطوبة ودرجة الحرارة في دلتا نهر اللؤلؤة؛ وعوامل معدل الكثافة السكانية وكثافة الطرق ومؤشر التغير الطبيعي للنباتات الخضراء ودرجة الحرارة ومعدل المسطحات المائية ونسبة سقوط الأمطار في الحدود بين مقاطعة يونان وميانمار أظهر أداءً جيداً في ما يتعلق بالدقة الإجمالية، مع تسجيل قيم 61 859.89 و 826.65 على معيار أكايكي للمعلومة، ما يفسر نسبة تباين تُقَدر بنسب 83.4% و 97.3% تباعاً. كما سبق، لدى العوامل الاجتماعية الاقتصادية تأثير على أوبئة حمى القش أقوى من تأثير العوامل البيئية في منطقة الدراسة. ومن بين هذه العوامل، كانت الكثافة السكانية (دلتا نهر اللؤلؤة) ومعدل الكثافة السكانية في الأرض العمرانية (الحدود بين مقاطعة يونان وميانمار) هما العاملين الاجتماعيين الاقتصاديين اللذين يفسران الاختلاف الأكبر في الأوبئة المستوطنة، بينما مؤشر التغير الطبيعي للنباتات الخضراء كان العامل البيئي الذي يفسر الخلاف الأكبر في المنطقتين. وإضافة إلى ذلك، أظهرت العوامل المشتركة (معدل الكثافة السكانية على الأرض العمرانية، ومؤشر التغير الطبيعي للنباتات الخضراء، ودرجة الحرارة) وفي هاتين المنطقتين تأثيرات مختلفة في الأوبئة المستوطنة.

الاستنتاجات: الأنماط الزمانية والمكانية لحمى القش في دلتا نهر اللؤلؤة والحدود بين مقاطعة يونان وميانمار تتأثر بالعوامل الاجتماعية والاقتصادية والبيئية، ويمكن أن تؤدي العوامل الاجتماعية والاقتصادية دوراً مهماً في أوبئة حمى القش في الحالات التي تكون فيها العوامل البيئية مناسبة ولا تختلف سوى قليلاً في أرجاء المنطقة. لذلك، يجب تخصيص مصادر الوقاية والضبط بالكامل من خلال الإشارة إلى الأنماط المكانية لعوامل التأثير الرئيسة من أجل ترسيخ أفضل لتدابير الوقاية والضبط ضد أوبئة حمى القش.

Translated from English version into Arabic by Aghareed Abdallah and Rami Alhames, through

TRANSLATORS
WITHOUT BORDERS

中国典型登革热疫区的时空格局及影响因素对比研究

Lan Zheng, Hong-Yan Ren, Run-He Shi

摘要
引言：作为全球最重要的蚊媒病毒性传染病，登革热近年来在中国的疫情形势日益严峻，对人民的健康造成了严重影响。因此，探究登革热疫情在中国典型疫区的时空格局及其主要影响因素是加强这些区域疫情有效防控的重要基础。
方法：以珠江三角洲和滇缅边境地区为例，本研究拟利用广义加性模型探析影响中国登革热典型疫区疫情时空格局差异的自然环境因素（NDVI、气温、降水、湿度）和社会经济因素（人口密度、道路密度、土地利用、GDP）。
结果：在 4km 和 3km 格网尺度，珠三角和滇缅边境地区的登革热疫情具有明显的空间分布特征，并分别在广佛地区、德宏州和西双版纳州等地区形成聚集。耦合“人口密度-城镇用地占比-NDVI-相对湿度-气温”共五个因素（珠三角）和“城镇用地占比-道路密度-NDVI-气温-水体占比-降水量”共六个因素（滇缅边境）的广义加性模型拟合效果较佳，Akaike Information Criterion 值分别为 61859.89 和 826.65，模型整体方差解释率分别达到 83.4%和 97.3%。在珠三角和滇缅边境地区，社会经济因素对疫情的影响要强于自然环境因素；其中，影响区域疫情的首位社会经济要素分别为人口密度（珠三角）和城镇用地占比（滇缅边境），而两个区域的首位自然环境因素均为 NDVI。此外，两个地区共有主要影响因素如城镇用地占比、NDVI 和气温等，对各区域疫情的影响也不尽相同。
结论：珠三角和滇缅边境地区的登革热疫情时空格局差异均会受到自然环境和社会经济因素的影响，而当区域自然环境因素适宜且内部差异不大的情况下，社会经济因素将在登革热流行中发挥更重要的作用，并且两个地区的影响机制亦有差异。本研究建议在合适的空间尺度上，结合区域疫情主要影响因素的格局特征有效调配防控资源，以加强登革热疫情防控。

Translated from English version into Chinese by Lan Zheng

Particularités spatio-temporelles et principaux facteurs d’influence des épidémies de dengue caractéristiques en Chine

Lan Zheng, Hong-Yan Ren, Run-He Shi

Résumé
Contexte : La dengue est la maladie infectieuse virale véhiculée par les moustiques la plus fréquente au monde. La Chine est touchée, depuis quelques années, par des épidémies de dengue de plus en plus sévères. Il est donc indispensable d’étudier les caractéristiques spatio-temporelles de ces épidémies et les facteurs susceptibles de les influencer dans des régions touchées, afin de renforcer les mesures de prévention et de contrôle de ces épidémies régionales et d’en accroître l’efficacité.
Méthodes : Un modèle additif généralisé (GAM) a été utilisé pour identifier les facteurs susceptibles d'influer sur la modélisation spatio-temporelle des épidémies dans les régions touchées par la dengue en Chine (par ex. le delta de la Rivière des Perles et la frontière entre le Yunnan et le Myanmar). Parmi ces facteurs, nous avons associé des facteurs environnementaux, tels que l’indice de végétation par différence normalisé (NDVI), la température, la pluviométrie et l’humidité, à des facteurs socio-économiques, tels que la densité de population (Pop), la densité du réseau routier, l’usage des terres et le produit intérieur brut.
Résultats : Les épidémies de dengue dans le delta de la Rivière des Perles et à la frontière entre

le Yunnan et le Myanmar présentent des variations considérables dans un maillage géographique de 4 km et 3 km, avec une agrégation spatiale significative dans les zones de Guangzhou-Foshan, Dehong et Xishuangbanna. Le GAM qui incluait les facteurs Pop/proportion de terres urbanisées/NDVI/humidité/température pour le delta de la Rivière des Perles, et proportion de terres urbanisées/densité du réseau routier/NDVI/température/proportion de paysages aquatiques/pluviométrie pour la frontière Yunnan-Myanmar a donné des résultats d’une bonne précision (valeurs du critère d’information d’Akaike de 61 859,89 et 826,65, montrant un écart total respectif de 83,4% et 97,3%). Les facteurs socio-économiques ont une plus forte influence sur les épidémies de dengue que les facteurs environnementaux dans la zone étudiée. Les variations des facteurs socio-économiques de densité de population (pour le delta de la Rivière des Perles) de proportion de terres urbanisées (pour la frontière Yunnan-Myanmar) sont celles qui ont eu l'impact le plus important sur les épidémies régionales, au même titre que le NDVI parmi les facteurs environnementaux. En outre, les facteurs communs à ces deux régions (proportion de terres urbanisées, NDVI et température) ont produit des effets différents sur les épidémies régionales.
Conclusions : La modélisation spatio-temporelle de la dengue dans le delta de la Rivière des Perles et à la frontière entre le Yunnan et le Myanmar varie selon des facteurs environnementaux et socio-économiques. Les facteurs socio-économiques peuvent avoir un impact significatif sur les épidémies de dengue lorsque les facteurs environnementaux s’y prêtent et ne varient que très peu à l’intérieur d’une zone. L’affectation des ressources pour la prévention et le contrôle devrait donc toujours s’appuyer sur la modélisation spatiale des principaux facteurs d’influence, afin d’accroître l’efficacité des mesures de prévention et de contrôle des épidémies de dengue.

Translated from English version into French by Suzanne Assenat and Estelle Rio, through

Пространственно-временные характеристики и первичные факторы, влияющие на типичные эпидемии лихорадки денге в Китае

Lan Zheng (Лань Чжэн), Hong-Yan Ren (Хун-Янь Жэнь), Run-He Shi (Жунь-Хэ Ши)

Аннотация

Краткая справка. Лихорадка денге (ЛД) − наиболее распространенная в мире передаваемая комарами вирусная инфекция. В последние годы все более серьезные эпидемии ЛД в Китае приводят к тяжелым последствиям для здоровья населения. Для принятия эффективных мер по предотвращению и контролю этих региональных эпидемий необходимо исследование пространственно-временных паттернов и потенциальных факторов, влияющих на эпидемии ЛД в типичных регионах.
Методы. Для определения потенциальных факторов, влияющих на пространственно-временные паттерны эпидемий, была использована обобщенная аддитивная модель. В исследовании были рассмотрены типичные регионы Китая, подверженные эпидемии ЛД,

такие как дельта реки Чжуцзян (ДРЧ) и граница провинции Юньнань и Мьянмы (ГЮМ). Были учтены факторы окружающей среды, включая стандартизованный индекс различий растительного покрова (СИРРП), температуру, количество осадков и влажность, в сочетании с социально-экономическими факторами, такими как плотность населения (ПН), плотность дорожной сети, землепользование и валовый внутренний продукт.
Результаты. При масштабах сетки 4 км и 3 км в регионах ДРЧ и ГЮМ была выявлена значительная пространственная изменчивость эпидемий ЛД, характеризующаяся выраженной пространственной кластеризацией в районах Гуанчжоу-Фошань, Дэхун и Сишуанбаньна. Обобщенные аддитивные модели были построены с учетом следующих факторов. Для региона ДРЧ учитывались соотношение ПН, уровень урбанизации (УУ), СИРРП, влажность и температура. Для региона ГЮМ принимались во внимание УУ, плотности дорожной сети, СИРРП, температура, соотношения воды и суши, а также количество осадков. О достаточной общей точности свидетельствовали значения информационного критерия Акаике 61859,89 и 826,65, что объясняет общую дисперсию, составляющую соответственно 83,4% и 97,3%. Очевидно, что в исследуемых регионах социально-экономические факторы имеют более сильное влияние на эпидемии ЛД, чем факторы окружающей среды. Было установлено, что социально-экономическими факторами, объясняющими наибольшую дисперсию в региональных эпидемиях, были ПН для ДРЧ и УУ для ГЮМ. При этом фактором, объясняющим наибольшую дисперсию в обоих регионах, был СИРРП. Кроме того, общие для обоих регионов факторы (УУ, СИРРП и температура) по-разному влияют на региональные эпидемии.
Выводы. На пространственно-временные паттерны ЛД в регионах ДРЧ и ГЮМ оказывают влияние факторы окружающей среды и социально-экономические факторы. При этом социально-экономические факторы могут играть значительную роль в эпидемиях ЛД там, где факторы окружающей среды благоприятны и незначительно отличаются на территории региона. Таким образом, для повышения эффективности мер по предотвращению и контролю эпидемий ЛД пространственные паттерны первичных факторов влияния должны в полной мере учитываться при распределении необходимых ресурсов.

Translated from English version into Russian by Anastasiia Dobrosynets and Natalia Potashnik, through

Características espaciotemporales y factores de influencia principales de las epidemias típicas de fiebre del dengue en China

Lan Zheng, Hong-Yan Ren, Run-He Shi

Resumen

Antecedentes: la fiebre del dengue (DF) es la enfermedad infecciosa viral transmitida por

mosquitos más común en el mundo, y las epidemias de DF cada vez más graves en China han afectado seriamente la salud de las personas en los últimos años. Por lo tanto, la investigación de los patrones espaciotemporales y los posibles factores de influencia de las epidemias de DF en las regiones típicas es fundamental para consolidar las medidas efectivas de prevención y control de estas epidemias regionales.

Métodos: Se utilizó un modelo aditivo generalizado (GAM) para identificar posibles factores contribuyentes que influyen en los patrones epidémicos espaciotemporales en las regiones epidémicas típicas de DF en China (por ejemplo, el delta del río Perla (PRD) y la frontera de Yunnan y Myanmar (BYM)). En términos de factores de influencia, se emplearon factores ambientales que incluyen el índice de vegetación de diferencia normalizada (NDVI), la temperatura, la precipitación y la humedad, junto con factores socioeconómicos, como la densidad de población (Pop), la densidad de la carretera, el uso de la tierra y el producto interior bruto.

Resultados: Las epidemias de DF en el PRD y BYM muestran variaciones espaciales destacadas en escalas de cuadrícula de 4 y 3 km, caracterizadas por un importante agrupamiento espacial en las áreas de Guangzhou-Foshan, Dehong y Xishuangbanna. El GAM que integró los factores de proporción de densidad de población/tierra urbana (ULR)/NDVI/humedad/temperatura para el PRD y los factores de proporción ULR/densidad de carretera/NDVI/temperatura/agua/tierra/precipitación para BYM tuvieron un buen desempeño en términos de precisión general, con valores del criterio de información de Akaike de 61 859,89 y 826,65, lo que explica una varianza total de 83,4% y 97,3%, respectivamente. Como se indicó, los factores socioeconómicos tienen una mayor influencia en las epidemias de DF que los factores ambientales en el área de estudio. Entre estos factores, Pop (PRD) y ULR (BYM) fueron los factores socioeconómicos que explican la mayor variación en las epidemias regionales, mientras que el NDVI fue el factor ambiental que explica la mayor variación en ambas regiones. Además, los factores comunes (ULR, NDVI y temperatura) en estas dos regiones mostraron efectos diferentes sobre las epidemias regionales.

Conclusiones: los patrones espaciotemporales de DF en el PRD y BYM están influenciados por factores ambientales y socioeconómicos, los factores socioeconómicos pueden jugar un papel importante en las epidemias de DF en casos en los que los factores ambientales son adecuados y difieren solo ligeramente en un área. Por lo tanto, los recursos de prevención y control deben asignarse por completo refiriéndose a los patrones espaciales de los factores de influencia principales para consolidar mejor las medidas de prevención y control para las epidemias de DF.

Translated from English version into Spanish by Xinia Arias and by Mayra León, through

TRANSLATORS
WITHOUT BORDERS
