# Supplementary material for: Spatiotemporal characteristics and primary influencing factors of typical dengue fever epidemics in China
Source: Infect Dis Poverty. 2019 Mar 28;8:24. doi: 10.1186/s40249-019-0533-9 (PMC6440137; doi:10.1186/s40249-019-0533-9)
Supplement: Supplementary file 2 — Pearson correlation analysis of variables in the PRD. (DOCX 19 kb) [file 40249_2019_533_MOESM2_ESM.docx]

Additional file 1 Pearson correlation analysis of variables in the PRD

|  | Pop | GDP | CLR | FLR | WLR | ULR | RLR | Hum | Pre | Temp | NDVI | Road |
| --- | --- | --- | --- | --- | --- | --- | --- | --- | --- | --- | --- | --- |
| Pop | 1 | 0.712^**^ | 0.324^**^ | -0.697^**^ | 0.192^**^ | 0.558^**^ | 0.418^**^ | -0.204^**^ | 0.126^**^ | 0.436^**^ | -0.616^**^ | 0.749^**^ |
| GDP |  | 1 | 0.060^**^ | -0.591^**^ | 0.210^**^ | 0.711^**^ | 0.449^**^ | -0.143^**^ | 0.152^**^ | 0.362^**^ | -0.597^**^ | 0.824^**^ |
| CLR |  |  | 1 | -0.628^**^ | 0.101^**^ | -0.130^**^ | 0.167^**^ | -0.193^**^ | 0.129^**^ | 0.427^**^ | -0.235^**^ | 0.071^**^ |
| FLR |  |  |  | 1 | -0.493^**^ | -0.425^**^ | -0.411^**^ | 0.114^**^ | -0.321^**^ | -0.654^**^ | 0.833^**^ | -0.599^**^ |
| WLR |  |  |  |  | 1 | 0.013 | 0.229^**^ | -0.043^**^ | 0.178^**^ | 0.342^**^ | -0.509^**^ | 0.200^**^ |
| ULR |  |  |  |  |  | 1 | 0.041^*^ | -0.079^**^ | 0.107^**^ | 0.240^**^ | -0.469^**^ | 0.803^**^ |
| RLR |  |  |  |  |  |  | 1 | -0.120^**^ | 0.100^**^ | 0.277^**^ | -0.333^**^ | 0.377^**^ |
| Hum |  |  |  |  |  |  |  | 1 | 0.720^**^ | -0.104^**^ | -0.031 | -0.188^**^ |
| Pre |  |  |  |  |  |  |  |  | 1 | 0.399^**^ | -0.367^**^ | 0.124^**^ |
| Temp |  |  |  |  |  |  |  |  |  | 1 | -0.598^**^ | 0.372^**^ |
| NDVI |  |  |  |  |  |  |  |  |  |  | 1 | -0.591^**^ |
| Road |  |  |  |  |  |  |  |  |  |  |  | 1 |
| Signif. codes: 0 ‘***’, 0.001 ‘**’, 0.01 ‘*’, 0.05; Pop: Population density; GDP: [Gross domestic product](https://baike.baidu.com/item/Gross%20Domestic%20Product); CLR: Cultivated land ratio; FLR: Forest land ratio; WLR: Water land ratio; ULR: Urban land ratio; RLR: Rural land ratio; Hum: Humidity; Pre: Precipitation; Temp: Temperature; NDVI: Normalized difference vegetation index; Road: Road density | | | | | | | | | | | | |
