# Supplementary material for: Spatiotemporal characteristics and primary influencing factors of typical dengue fever epidemics in China
Source: Infect Dis Poverty. 2019 Mar 28;8:24. doi: 10.1186/s40249-019-0533-9 (PMC6440137; doi:10.1186/s40249-019-0533-9)
Supplement: Supplementary file 3 — Pearson correlation analysis of variables in the BYM. (DOCX 18 kb) [file 40249_2019_533_MOESM3_ESM.docx]

Additional file 2 Pearson correlation analysis of variables in the BYM

|  | Pop | GDP | CLR | FLR | WLR | ULR | RLR | Hum | Pre | Temp | NDVI | Road |
| --- | --- | --- | --- | --- | --- | --- | --- | --- | --- | --- | --- | --- |
| Pop | 1.000 | 0.762^**^ | 0.563^**^ | -0.378^**^ | 0.142^**^ | 0.717^**^ | 0.279^**^ | -0.269^**^ | -0.234^**^ | 0.151^**^ | -0.377^**^ | 0.507^**^ |
| GDP |  | 1.000 | 0.214^**^ | -0.153^**^ | 0.104^**^ | 0.684^**^ | 0.322^**^ | -0.102^**^ | -0.070^**^ | 0.113^**^ | -0.210^**^ | 0.452^**^ |
| CLR |  |  | 1.000 | -0.572^**^ | 0.092^**^ | 0.067^**^ | 0.158^**^ | -0.294^**^ | -0.271^**^ | 0.161^**^ | -0.377^**^ | 0.232^**^ |
| FLR |  |  |  | 1.000 | -0.134^**^ | -0.081^**^ | -0.126^**^ | 0.274^**^ | 0.274^**^ | -0.177^**^ | 0.385^**^ | -0.253^**^ |
| WLR |  |  |  |  | 1.000 | 0.072^**^ | 0.049^**^ | -0.186^**^ | -0.147^**^ | 0.154^**^ | -0.172^**^ | 0.161^**^ |
| ULR |  |  |  |  |  | 1.000 | 0.059^**^ | -0.040^**^ | -0.021 | 0.056^**^ | -0.154^**^ | 0.393^**^ |
| RLR |  |  |  |  |  |  | 1.000 | -0.060^**^ | -0.050^**^ | 0.061^**^ | -0.094^**^ | 0.224^**^ |
| Hum |  |  |  |  |  |  |  | 1.000 | 0.873^**^ | -0.346^**^ | 0.216^**^ | -0.144^**^ |
| Pre |  |  |  |  |  |  |  |  | 1.000 | 0.007 | 0.318^**^ | -0.124^**^ |
| Temp |  |  |  |  |  |  |  |  |  | 1.000 | 0.161^**^ | 0.177^**^ |
| NDVI |  |  |  |  |  |  |  |  |  |  | 1.000 | -0.241^**^ |
| Road |  |  |  |  |  |  |  |  |  |  |  | 1.000 |
| Signif. codes: 0 ‘***’, 0.001 ‘**’, 0.01 ‘*’, 0.05; Pop: Population density; GDP: [Gross domestic product](https://baike.baidu.com/item/Gross%20Domestic%20Product); CLR: Cultivated land ratio; FLR: Forest land ratio; WLR: Water land ratio; ULR: Urban land ratio; RLR: Rural land ratio; Hum: Humidity; Pre: Precipitation; Temp: Temperature; NDVI: Normalized difference vegetation index; Road: Road density | | | | | | | | | | | | |
